# Modulation of Gut Bacterial and Fungal Microbiota in Fibromyalgia Patients Following a Carb-Free Oloproteic Diet: Evidence for Candida Suppression and Symptom Improvement

**DOI:** 10.3390/microorganisms13092069

**Published:** 2025-09-05

**Authors:** Giuseppe Castaldo, Maria D’Elia, Mariagrazia De Prisco, Veronica Folliero, Carmen Marino, Annamaria D’Ursi, Gianluigi Franci, Luca Rastrelli

**Affiliations:** 1 NutriKeto_LAB Unisa, “San Giuseppe Moscati” National Hospital (AORN), Contrada Amoretta, 83100 Avellino, Italy; giuseppecastaldo@yahoo.it; 2 Department of Pharmacy, University of Salerno, Via Giovanni Paolo II, 132, 84084 Salerno, Italy; mdelia@unisa.it (M.D.); cmarino@unisa.it (C.M.); dursi@unisa.it (A.D.); 3 National Biodiversity Future Center (NBFC), 90133 Palermo, Italy; 4 Department of Earth and Marine Sciences, University of Palermo, 90133 Palermo, Italy; 5 Department of Medicine, Surgery and Dentistry “Scuola Medica Salernitana”, University of Salerno, 84081 Baronissi, Italy; depriscomariagrazia22@gmail.com (M.D.P.); vfolliero@unisa.it (V.F.)

**Keywords:** fibromyalgia, gut microbiota, ketogenic diet, oloproteic diet, fecal samples, candida albicans, 16S/18S rRNA sequencing

## Abstract

Fibromyalgia (FM) is a complex chronic syndrome characterized by widespread pain, fatigue, and gastrointestinal complaints. Clinical observations and preliminary metabolomic data suggest a possible link between symptom severity and intestinal dysbiosis, including fungal overgrowth. This study investigates whether a carb-free oloproteic diet can beneficially modulate the gut microbiota in FM patients. Thirty-four female patients with diagnosed FM were enrolled in a controlled, parallel-arm nutritional intervention. Group FM1 (n = 22) followed a 45-day carb-free oloproteic diet followed by a 45-day low-glycemic (LOGI) diet. Group FM2 (n = 12) received a continuous LOGI diet for 90 days. They were collected at baseline (T0), after 45 days (T45), and at 90 days (T90). Microbial profiles were analyzed by 16S and 18S rRNA gene sequencing to assess bacterial and fungal composition. In FM1, the oloproteic phase led to a marked reduction in fungal abundance (Ascomycota) and an increase in butyrate-producing bacteria such as Faecalibacterium and Roseburia. These changes were partially reversed after the LOGI phase. In FM2, no significant microbiota shifts were observed. Clinical improvements paralleled microbiota modulation only in FM1. The carb-free oloproteic diet may support gut microbial rebalancing in FM, particularly through transient suppression of fungal overgrowth. These findings support further investigation into nutritional strategies targeting dysbiosis in FM management.

## 1. Introduction

Fibromyalgia (FM) is a complex, multifactorial syndrome with a strong female predominance, characterized by chronic widespread pain, fatigue, non-restorative sleep, cognitive impairments (such as poor memory and concentration), mood disturbances, and somatic symptoms, including gastrointestinal discomfort [1,2,3,4]. Classified among rheumatologic disorders, FM remains clinically challenging due to the lack of definitive diagnostic markers and the limited efficacy of current pharmacological treatments, which often provide only partial or transient relief. Recent scientific evidence has increasingly recognized inflammation as a key factor in the etiopathogenesis of fibromyalgia (FM) [4,5]. Chronic inflammatory diseases have been strongly associated with gut microbiota imbalances, and several studies have documented significant dysbiosis in FM patients. These microbiome alterations suggest a possible role for microbes in disease progression and modulation of symptom severity, particularly through mechanisms related to immune regulation, intestinal permeability, and mitochondrial stress [6,7].

The human gut microbiome constitutes an extremely complex and diverse ecosystem of microorganisms, essential for maintaining host homeostasis and influencing numerous pathophysiological processes [8]. Growing evidence has also linked alterations in the gut microbiota to the pathogenesis of multiple chronic conditions, including fibromyalgia [1,9]. While most studies have focused on bacterial dysbiosis, the contribution of the intestinal fungal community (mycobiota) has remained largely unexplored. In clinical practice, many fibromyalgia patients report symptoms consistent with a microbial imbalance, such as bloating, feeding intolerance, and impaired intestinal function, which may reflect underlying fungal overgrowth [10,11]. Among the fungal species, *Candida albicans* is of particular interest. Although normally a commensal organism, Candida can transform into an aggressive hyphal form, capable of breaching mucosal barriers and producing toxins and metabolites that can contribute to systemic symptoms such as fatigue, increased pain sensitivity, cognitive impairment, and neuroinflammation [12,13]. Importantly, estrogen signaling has been shown to promote Candida growth and hyphal transformation, which may partially explain the higher incidence and symptom burden in women [14]. Diet is a primary modulator of the composition and function of the gut microbiota. Various dietary interventions, including low-FODMAP and ketogenic diets, have been associated with reduced pain and improved quality of life in fibromyalgia patients [15,16]. Through over 30 years of clinical experience, we have observed that fibromyalgia symptoms, including fatigue, pain, and digestive discomfort, often improve following a carbohydrate-free, holoprotein (ketogenic) diet protocol. These improvements were largely maintained during the subsequent low-glycemic index (LOGI) phase, albeit with modest clinical regression, an expected outcome given the reintroduction of carbohydrates. Nonetheless, the LOGI diet represents a feasible and sustainable option for transitioning from a ketogenic protocol, which, while effective, is not suitable for long-term adherence. In two previous ethically approved clinical trials, conducted in collaboration with rheumatologists at a public hospital, we demonstrated that the holoprotein nutritional protocol significantly improved clinical symptoms and metabolic profiles in patients with fibromyalgia and that the subsequent LOGI diet proved to be an effective and sustainable maintenance strategy. In this context, our study evaluates a clinically validated dietary protocol, comprising a carbohydrate-free holoprotein phase followed by a low-glycemic index maintenance phase, to assess its impact on intestinal bacterial and fungal communities and its potential to modulate inflammation and fibromyalgia-related symptoms. Specifically, we investigated whether the observed clinical improvements could be associated with changes in the gut microbiota composition, particularly in relation to fungal overgrowth. Using 16S and 18S rRNA sequencing of fecal samples collected at three timepoints (T0, T45, and T90), we compared the effects of a holoprotein-LOGI protocol (FM1) versus a LOGI-only regimen (FM2) in female patients with FM, to explore the role of dietary strategies in rebalancing the microbiota and alleviating FM symptoms. The current study builds on two previous clinically validated studies, conducted in collaboration with rheumatologists and approved by the relevant Ethics Committee, which demonstrated significant symptomatic improvements in fibromyalgia patients who followed this nutritional protocol. Several limitations in previous research necessitated the present study. Although a growing body of evidence has linked alterations in the gut microbiota to fibromyalgia, most of these studies have focused on bacterial imbalances, while the role of the intestinal fungal community has remained largely unexplored. Furthermore, although some research has shown that dietary interventions such as low-FODMAP and ketogenic diets can help reduce pain and improve quality of life in fibromyalgia patients, there is still a gap in our understanding of how these diets specifically affect intestinal bacterial and fungal populations. This study aims to fill this gap by evaluating how a dietary protocol with a carbohydrate-free holoprotein phase followed by a LOGI maintenance phase influences both bacterial and fungal communities and whether these changes are associated with the observed clinical improvements in fibromyalgia-related symptoms.

## 2. Materials and Methods

### 2.1. Study Design and Participants

This study enrolled 34 female patients aged 30–65 years with a confirmed diagnosis of fibromyalgia according to the American College of Rheumatology (ACR) criteria. Participants were recruited from a rheumatology outpatient clinic and provided written informed consent. The protocol was approved by the local Ethics Committee and conducted under the Declaration of Helsinki. Patients were assigned to two dietary intervention groups. The FM1 group (n = 22) underwent a two-phase nutritional protocol consisting of a carbohydrate-free oloproteic diet for the initial 45 days, followed by a low-glycemic index (LOGI) diet for an additional 45 days. On the other side, the FM2 group (n = 12) received a continuous LOGI diet for the entire 90-day period. Clinical assessments, biochemical analyses, and fecal sample collections were conducted at three timepoints: baseline (T0), after 45 days (T45), and after 90 days (T90). This study aimed to test whether a carbohydrate-free holoprotein diet could effectively modulate the gut microbiota in patients with fibromyalgia, potentially contributing to improved clinical outcomes.

### 2.2. Dietary Intervention

In this study, 34 participants diagnosed with fibromyalgia (FM) were divided into two groups to evaluate the effects of targeted nutritional interventions on both clinical outcomes and gut microbiota composition. The FM1 group, composed of 22 individuals, followed a two-phase dietary regimen. The very-low-carbohydrate ketogenic diet (VLCKD) was based on the Blackburn protocol, also known as modified protein-sparing fasting (PSMF) [17]. This approach involved a near-total elimination of carbohydrates, limiting intake to approximately 10 g per day, primarily from selected vegetables and dairy products. Protein intake was 1.4 g per kg of ideal body weight, calculated using the Lanzola formula [18], and came from lean sources such as meat and fish. To preserve lean body mass and stimulate growth hormone secretion, participants were supplemented with whey protein enriched with essential amino acids and 10 g/day of hydrolyzed collagen. Fat intake varied between 45 and 100 g/day and was inversely proportional to body mass index (BMI), with the main sources being extra virgin olive oil and coconut oil [19]. Raw or cooked vegetables were allowed for lunch and dinner, excluding those with more than 1.5 g of carbohydrates per 100 g [20]. The protocol also included additional measures to ensure safety and efficacy, such as phytotherapeutic agents, alkalizing mineral salts, and special salts [21]. Participants were required to drink at least 2 L of alkaline mineral water per day and were given Triphala to prevent constipation. Alcohol and stimulant drinks were prohibited or limited [22]. The comparison group, consisting of 12 participants, followed a low glycemic index (LOGI) diet for the entire study. This diet, based on a Mediterranean model, included a total caloric intake between 20 and 35 kcal/kg of ideal body weight. Carbohydrate intake was less than 50% of total daily calories, from low glycemic index sources. Gluten and lactose intake was minimized, and pasta was allowed only once a week. Protein was maintained at 1.2 g/kg of ideal body weight, and fat intake, between 45 and 100 g/day, included monounsaturated and MCT-rich fats. Participants were advised to drink a minimum of 2 L of low-sodium mineral water each day, with alcohol strictly prohibited [23,24,25].

### 2.3. Fecal Sample Collection and Storage

Fecal samples were collected by participants using sterile, DNA/RNA-free containers to prevent external contamination. Samples were promptly transported to the laboratory and stored at −80 °C to maintain DNA integrity until processing. Before DNA extraction, each sample was thoroughly homogenized, and 250 mg of fecal material was used, providing sufficient biomass for downstream microbiota analyses. Microbial DNA was isolated using the QIAamp Fast DNA Stool Mini Kit (Qiagen, Hilden, Germany) according to the manufacturer’s protocol. DNA concentration and purity were initially assessed using a NanoDrop 2000 spectrophotometer (Thermo Fisher Scientific, Waltham, MA, USA) and subsequently validated by fluorometric quantification with a Qubit 4 Fluorometer and the dsDNA High Sensitivity Assay Kit (Invitrogen, Thermo Fisher Scientific, Waltham, USA).

### 2.4. Library Construction and Sequencing

For bacterial community profiling, the V3–V4 hypervariable regions of the 16S rRNA gene were amplified using the primer pair 341F (5′-CCTACGGGNGGCWGCAG-3′) and 805R (5′-GACTACHVGGGTATCTAATCC-3′). For fungal communities, the V9 region of the 18S rRNA gene was targeted using primers 1380F (5′-GATGAAGAACGYAGYRAA-3′) and 1510R (5′-TCCTCCGCTTWTTGWTWTGC-3′). PCR amplifications were performed using Platinum SuperFi II DNA Polymerase (Thermo Fisher Scientific, Waltham, USA) and dNTP mix to ensure high-fidelity amplification. Amplicons were purified with AMPure XP magnetic beads (Beckman Coulter, Brea, CA, USA) to remove residual primers, dimers, and contaminants. Sequencing libraries were prepared using the Nextera XT DNA Library Preparation Kit (Illumina Inc., San Diego, CA, USA). Libraries were quantified using the Qubit 4 Fluorometer (Invitrogen, Thermo Fisher Scientific, Waltham, USA), pooled in equimolar concentrations, and sequenced on the Illumina MiSeq platform using 2 × 300 bp paired-end reads and the MiSeq v3 600-cycle reagent kit (MS-102-3003) (Illumina, San Diego, USA), according to the manufacturer’s protocol.

### 2.5. Bioinformatics and Statistical Analysis

Raw sequencing data underwent quality filtering and processing using QIIME2 software (version 2023.2). Operational taxonomic units (OTUs) were clustered at 97% sequence similarity, with taxonomic assignments performed using the Greengenes database for bacterial 16S rRNA gene sequences and the SILVA database for fungal 18S rRNA gene sequences. Beta diversity was evaluated using Bray–Curtis dissimilarity and visualized through Principal Coordinates Analysis (PCoA). Differential abundance analyses were conducted employing LEfSe (Linear Discriminant Analysis Effect Size) and ANCOM (Analysis of Composition of Microbiomes) to identify taxa significantly associated with experimental groups. Statistical significance was defined at *p* < 0.05. All downstream statistical analyses and graphical visualizations were performed using R software (version 4.2.1) and GraphPad Prism (version 9.5). Laboratory workflows were executed within a certified molecular microbiology facility under stringent sterile and quality-controlled conditions.

## 3. Results

### 3.1. Gut Microbiota Dynamics Across Dietary Interventions

**Figure 1:**
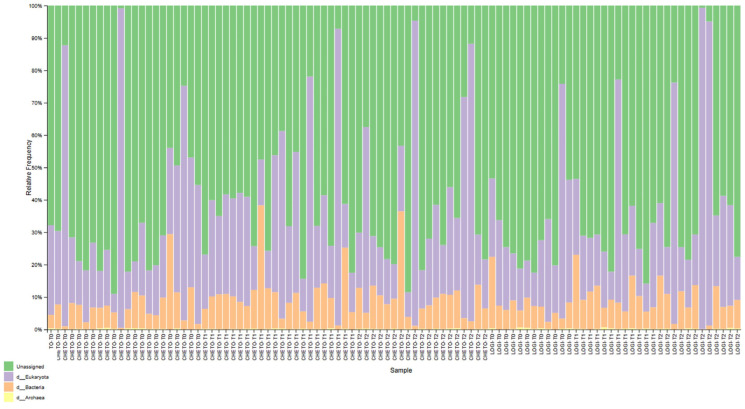
Gut bacterial composition across timepoints in FM1 and FM2 patients. Stacked bar plots show the relative abundance of bacterial taxa (phylum level) in fecal samples collected at baseline (T0), after 45 days (T1), and after 90 days (T2). Samples are grouped by treatment arm: FM1 (oloproteic diet followed by LOGI) and FM2 (LOGI only). Each bar represents an individual subject. The OD + LOGI group exhibited a progressive decrease in fungal-related taxa and an increase in beneficial Firmicutes over time, whereas the LOGI-only group displayed more heterogeneous microbial profiles and higher proportions of potentially pro-inflammatory taxa.

**Figure 2:**
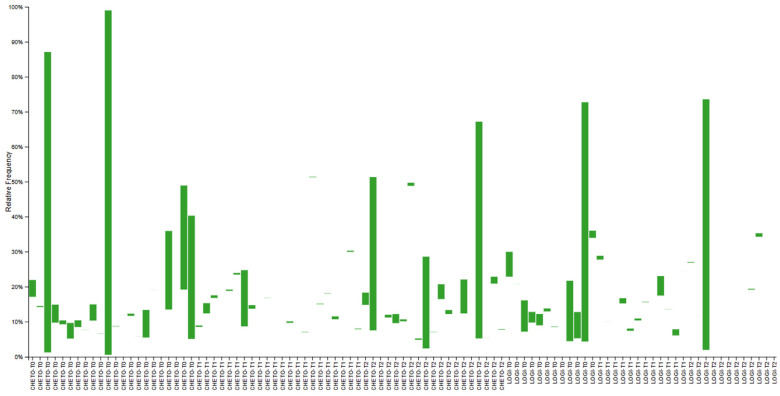
Relative abundance of the fungal phylum Ascomycota across timepoints in FM1 and FM2 patients. Bar plot illustrates the relative abundance (%) of Ascomycota in fecal samples from fibromyalgia patients at baseline (T0), after 45 days (T1), and after 90 days (T2). Subjects in the FM1 group (oloproteic diet followed by LOGI) showed a marked reduction in Ascomycota levels at T1, followed by a partial rebound at T2. In contrast, patients in the FM2 group (LOGI-only diet) maintained consistently low Ascomycota abundance throughout the 90-day intervention, suggesting that the LOGI diet provides a stable environment that limits fungal overgrowth.

Gut microbiota profiling via 16S rRNA sequencing revealed significant temporal and group-specific shifts in bacterial community structure across the two dietary interventions (Figure 1). In FM1 patients, who underwent an oloproteic diet (OD) followed by the LOGI diet, a progressive enrichment of beneficial Firmicutes was observed, accompanied by a concomitant reduction in potentially pro-inflammatory phyla such as Proteobacteria and fungal-related sequences from baseline (T0) through T45 to T90. Conversely, FM2 patients, subjected solely to the LOGI diet, displayed more heterogeneous microbial patterns characterized by less pronounced compositional shifts and persistently elevated levels of Bacteroidota and Proteobacteria, indicative of a relatively stable yet potentially dysbiotic community. This pattern was further supported by analysis of the fungal phylum Ascomycota through 18S rRNA gene data (Figure 2). FM1 participants showed a marked decrease in Ascomycota after the ketogenic phase (T45), with a moderate resurgence observed during the subsequent LOGI phase (T90). In contrast, FM2 patients maintained consistently stable Ascomycota levels throughout the study period, suggesting that the LOGI diet alone does not exert an antifungal effect, whereas the OD induces a more pronounced modulation of fungal taxa. Collectively, these findings highlight that the dual-diet protocol in FM1 led to a more dynamic and beneficial restructuring of both the bacterial and fungal components of the gut microbiome, whereas the LOGI-only approach in FM2 resulted in more static microbial patterns, potentially reflective of limited ecological perturbation or resilience to dietary modulation.

### 3.2. Beta-Diversity and Community Structure Shifts

**Figure 3:**
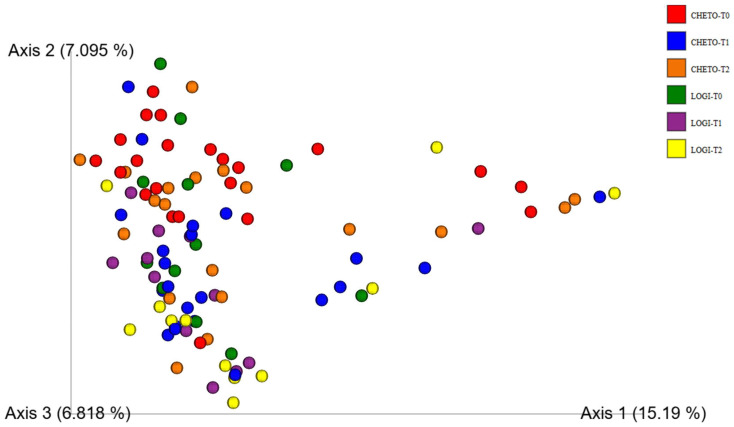
Principal Coordinates Analysis (PCoA) of gut bacterial β-diversity in FM patients during dietary intervention. PCoA based on Bray–Curtis dissimilarity index shows the distribution of gut bacterial communities across six timepoints: FM1-T0 (red), FM1-T45 (blue), FM1-T90 (orange), FM2-T0 (green), FM2-T45 (purple), and FM2-T90 (yellow). Although the global dataset displays high dispersion, trends suggest a temporal restructuring of the gut microbiota, particularly in FM1 patients, where T45 and T90 samples progressively diverge from baseline and partially overlap with FM2 samples at T90. The first three axes explained 15.19%, 7.10%, and 6.82% of the variance.

Beta-diversity analysis based on Bray–Curtis dissimilarity confirmed that gut microbial communities followed different dynamics over time and in relation to diet. When looking at the complete dataset (Figure 3), a rather complex picture emerges: the data points appear dispersed, a sign of the high individual variability and the differences between the two dietary protocols. Despite this dispersion, an evolutionary path is evident, especially in the FM1 group, whose samples progressively move from the basal phase (T0, red) to the intermediate phase after the holoprotein diet (T45, blue), and finally to the phase following the transition to the LOGI diet (T90, orange). This trend suggests that the dual nutritional strategy had a profound and sequential impact on microbiota. Interestingly, at the end of the study, the microbial profiles of the FM1 group at T90 tended to partially overlap with those of the FM2 group (T90, yellow), indicating some convergence despite the initially different dietary pathways. It is plausible that the reintroduction of carbohydrates in the LOGI phase favored the emergence of more similar microbial configurations, while the FM1 group showed a more dynamic restructuring process, likely triggered by the initial ketogenic intervention. The first three PCoA axes explained 15.19%, 7.10%, and 6.82% of the variance, values reflecting moderate separation of the microbial communities.

**Figure 4:**
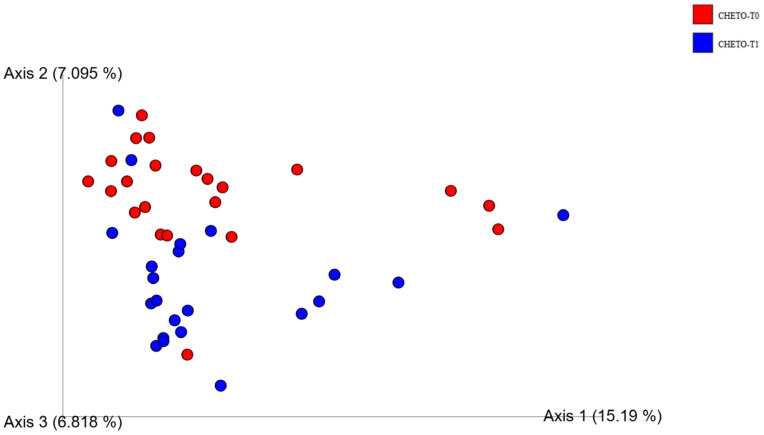
Targeted PCoA analysis of gut bacterial β-diversity in FM1 patients between baseline and post-ketogenic phase. Principal Coordinates Analysis (PCoA) based on Bray–Curtis dissimilarity index shows clearer clustering of gut bacterial communities in FM1 before (T0, red) and after 45 days of ketogenic intervention (T45, blue). The separation between the two timepoints along the first three axes (15.19%, 7.10%, and 6.82%) highlights the strong modulatory effect of the ketogenic diet.

To further clarify the effect of the ketogenic diet, a targeted analysis was conducted, limited to samples from the FM1 group at baseline and after 45 days (Figure 4). In this restricted comparison, the separation between the two timepoints was much more evident, highlighting the clear impact of the holoprotein intervention on the microbiota structure. The distinction between pre- and post-OD samples reinforces the idea that a ketogenic diet can rapidly and profoundly reshape the intestinal ecosystem. These findings are consistent with the literature, which reports that low-carbohydrate, high-protein diets can exert selective pressure, favoring microorganisms better suited to metabolizing proteins and fats and reducing carbohydrate fermenters, which are often associated with inflammatory processes. Overall, β-diversity analyses not only confirm the dynamic and diet-dependent nature of microbial restructuring but also suggest that a two-phase approach, such as that followed in the FM1 group, can induce more profound and potentially beneficial changes than a single intervention, such as the LOGI diet alone.

### 3.3. Eukaryotic Microbiome Complexity

More detailed analysis of the eukaryotic component of gut microbiome was achieved through 18S rRNA analysis, which revealed a highly complex and dynamic profile of fungal communities across study groups and timepoints (Figure 3). As expected, members of the phyla Ascomycota and Basidiomycota predominated throughout the dataset, representing the main fungal constituents of the intestinal mycobiome in both FM1 and FM2 cohorts. However, besides these dominant taxa, the analysis revealed substantial inter-individual variability and considerable temporal fluctuations in several other eukaryotic phyla, including Mucoromycota, Apicomplexa and Arthropoda. These variations were notably marked in the FM1 group, mainly during the holoprotein diet (OD) phase, suggesting that this specific dietary intervention could exert broader and more profound modulatory impacts on the eukaryotic gut microbiome than the LOGI diet alone. For instance, short-lived increases in Mucoromycota at T45 were noted in some FM1 individuals, which could reflect a change in environmental niches or nutritional substrates during the ketogenic phase. Occasional presences of Apicomplexa were noted in FM1 but remained virtually undetectable in FM2. Arthropod-associated sequences, although less abundant, also revealed a variable presence, showing the broader ecological impact of diet composition on eukaryotic gut microbes. These findings highlight the complexity of the human eukaryotic microbiome and suggest that dietary interventions could influence not only bacterial communities, but also less studied fungal and protist components. The marked, diet-dependent changes noted in FM1 support the hypothesis that ketogenic diets could serve as more pronounced ecological disruptions, reshaping eukaryotic niches in the gut ecosystem.

### 3.4. Enhanced Beta-Diversity Resolution with Updated Analysis

**Figure 5:**
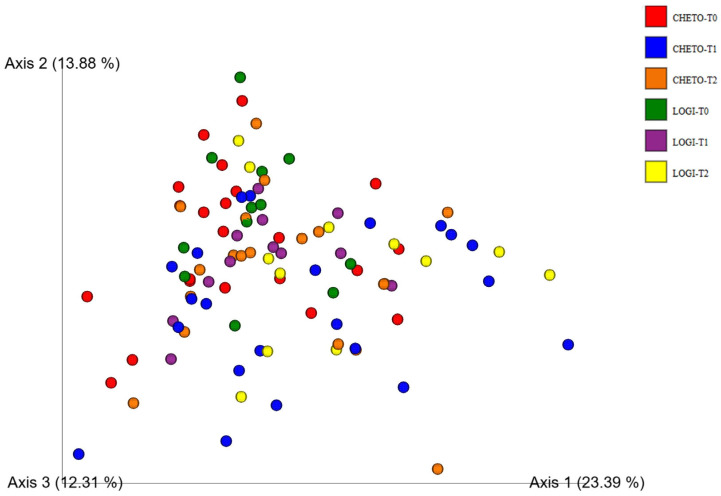
Principal Coordinates Analysis (PCoA) of gut bacterial β-diversity based on 16S rRNA sequencing. PCoA plot based on Bray–Curtis dissimilarity index illustrates the clustering of gut bacterial communities across the six timepoints: FM1-T0 (red), FM1-T45 (blue), FM1-T90 (orange), FM2-T0 (green), FM2-T45 (purple), and FM2-T90 (yellow). The variance explained by the first three axes was 23.39%, 13.88%, and 12.31%, respectively. Although considerable inter-individual variability was observed, a general trend of partial separation over time, particularly between baseline and T90, suggests a gradual microbiota shift influenced by dietary intervention. These findings reinforce the impact of nutritional modulation on gut bacterial community structure in FM patients.

The broader β-diversity patterns noted in the initial analysis were further verified by a second Principal Coordinate Analysis (PCoA), which included updated 16S rRNA sequencing data and higher resolution ordination axes (Figure 5). This improved analysis clarified a greater proportion of the total variance in microbial community composition, with axis 1 elucidating 23.39%, axis 2 13.88% and axis 3 12.31%. The resulting PCoA plot revealed a clear variation in microbial profiles as a function of time, particularly evident when comparing basal (T0) and final (T90) timepoints in both FM1 and FM2 groups. In the FM1 cohort, which followed a sequential holoprotein dietary regimen and LOGI, the participants showed a consistent trajectory between timepoints, advancing progressively along the sorting space. This trend suggested a structured and gradual rebalancing of the gut microbiota, with distinct groupings at each intervention point (T0, T45, T90). These directional shifts affirm the hypothesis that dual-diet intervention exerts a cumulative and temporally coordinated impact on microbial ecology.

In contrast, group FM2, subjected exclusively to the LOGI diet, also showed a temporal separation between timepoints, but with a greater dispersion and overlapping of clusters, revealing greater inter-individual variability and less cohesive community changes. Whilst both groups were affected by restructuring of the microbiota over time, the changes in FM2 were finer and more variable, supporting the hypothesis that the holoprotein phase in FM1 could serve as a stronger perturbation, triggering the gut ecosystem for post-event stabilization during re-feeding. These results highlight the dietary impact on the gut microbiome and underline the importance of sequencing interventions. The results also stress the utility of high-resolution β diversity analyses to capture the nuances of microbial community dynamics over time.

### 3.5. Distinct Clustering Following Oloproteic Diet in FM1

**Figure 6:**
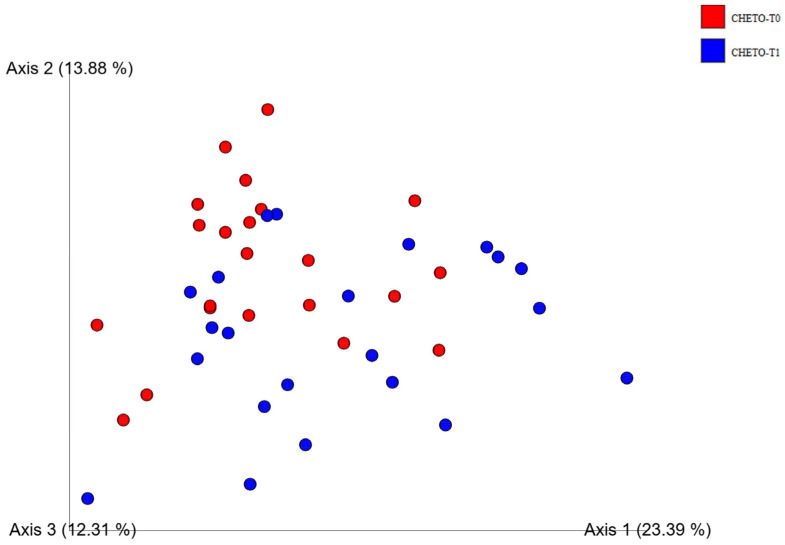
PCoA analysis of gut bacterial β-diversity before and after ketogenic intervention (FM1 group). Principal Coordinates Analysis (PCoA) based on Bray–Curtis dissimilarity illustrates shifts in gut bacterial community composition between FM1-T0 (red) and FM1-T45 (blue), corresponding to the baseline and post-ketogenic diet phase, respectively. The three axes explained 23.39% (axis 1), 13.88% (axis 2), and 12.31% (axis 3) of the total variance. A partial separation between the two timepoints is evident, supporting the hypothesis that the ketogenic dietary phase induces modifications in microbial β-diversity in FM patients.

Solely targeting the FM1 group, comparison of timepoints before the diet (T0) and after the holoprotein diet (T45) revealed a distinct grouping pattern in the PCoA graph (Figure 6), strongly indicative of diet-induced changes in gut microbial β-diversity. This divide highlights the major impact of the holoprotein (OD) phase on microbial community structure, indicating that even a moderately short 45-day intervention is sufficient to reconfigure the gut ecosystem in a visible trend and consistent manner. Although some overlap continued, showing the inter-individual variability and endurance of some microbial taxa, axis 1 consistently emerged as the most selective dimension, accounting for 23.39% of the total variance. This axis was captured with rigor, tracking the main trajectory of microbial adaptation caused by the ketogenic phase of the diet. Post-OD sampling units were characterized by a reduction in taxa associated with pro-inflammatory profiles, such as Proteobacteria, and a parallel enrichment of beneficial groups, mainly within Firmicutes and Actinobacteria, often related to improved metabolic and immune homeostasis. The impact of this alteration, both in terms of distance within the PCoA space and taxonomic restructuring, affirmed the hypothesis that the holoprotein intervention applied selective pressure to the gut microbiota, perhaps altering nutrient availability, host-derived metabolites or gut pH. Resuming the LOGI diet after this phase seemed to consolidate some of these microbial shifts rather than reverting them completely, indicating a lasting impact of the OD phase. These results highlighted the adaptive nature of the gut microbiota in response to diet and confirmed that even short-term nutritional strategies can cause significant compositional and structural changes.

### 3.6. Phylum-Level Compositional Changes

**Figure 7:**
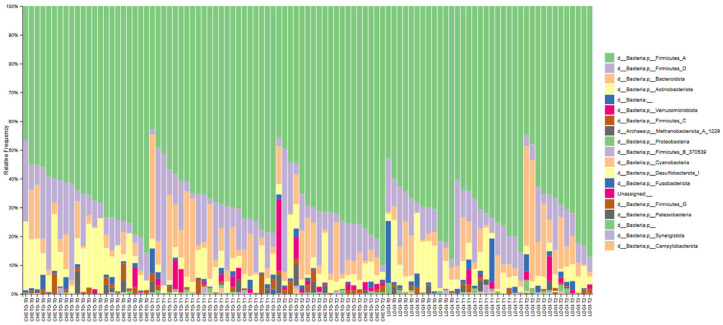
Taxonomic composition of gut bacterial communities across dietary intervention timepoints. Stacked bar plots represent the relative abundance of bacterial taxa (16S rRNA sequencing) in fecal samples across six experimental conditions: FM1-T0 (oloproteic diet, OD), FM1-T45 (OD), FM1-T90 (LOGI), FM2-T0 (LOGI), FM2-T45 (LOGI), and FM2-T90 (LOGI). Each bar corresponds to a single individual sample. The figure highlights a pronounced interindividual variability and the dominance of Firmicutes (green), Bacteroidota (purple), and other major phyla. Temporal fluctuations in the relative abundance of taxa such as Actinobacteria, Proteobacteria, and Verrucomicrobiota suggest microbial community shifts in response to the ketogenic and LOGI dietary phases.

The phylum-level data provided a comprehensive overview of the compositional dynamics of gut bacterial communities during dietary interventions (Figure 7). At all phases and in all groups, Firmicutes represented the dominant phylum, highlighting their central role in the human gut microbiome. However, the relative abundance of Firmicutes changed differentially between the two intervention groups, with FM1 (a holoprotein diet followed by LOGI) showing a more pronounced and dynamic variation than the relatively stable patterns observed in FM2 (LOGI only). In FM1, an evident increase in Actinobacteria was noted during the re-feeding phase (LOGI), particularly at T90, indicating the expansion of potentially beneficial taxa, such as Bifidobacterium, which is often associated with improved gut barrier function, reduced inflammation, and metabolic regulation. During the ketogenic OD phase, we observed a transient but significant increase in Verrucomicrobiota. Although our analyses do not allow the identification of specific species, this phylum includes taxa such as *Akkermansia muciniphila*, which are known to degrade mucin. This suggests a potential role for Verrucomicrobiota members in intestinal adaptation to low-carbohydrate, high-protein conditions, without implying that the specific species was directly detected. These changes indicate an adaptable and reactive microbial ecosystem, able to adapt its composition in response to the different nutritional pressures imposed by ketogenic and moderate-carbohydrate diets. In contrast, FM2 samples showed greater taxonomic stability at all phase points, indicating the more gradual and less intense impact of a LOGI diet administered over the entire intervention period. Although this group revealed less interindividual variability, the microbial profile was characterized by persistently lower levels of Actinobacteria and Verrucomicrobiota, taxa often considered indicators of a healthy microbiota. Furthermore, Proteobacteria and Bacteroidetes persisted relatively high in FM2, particularly at T90, potentially indicating a less favorable or slower transition to eubiotic equilibrium. In sum, this compositional pattern suggests that the sequential dietary strategy applied in FM1, which begins with a restrictive oligoprotein phase and is followed by a LOGI reintroduction phase, exerted a more marked and beneficial modulatory effect on the composition of the gut microbiota. This two-phase intervention appears to aid an expanded taxonomic profile in phyla commonly associated with metabolic and immunological benefits, showing its potential therapeutic value in modulating gut microbial ecology.

## 4. Discussion

This study provides compelling evidence that targeted dietary interventions can significantly modulate gut microbiota in patients with fibromyalgia (FM). Using 16S and 18S rRNA gene sequencing, we captured bacterial and eukaryotic community dynamics in a 90-day nutritional protocol consisting of either a LOGI diet alone (FM2) or a combined ketogenic-LOGI diet (FM1). The holoprotein (ketogenic) phase in the FM1 group induced a rapid and marked shift in bacterial β-diversity, as evidenced by PCoA analyses (Figure 3, Figure 4 and Figure 7). Notably, samples collected after 45 days of OD intervention (FM1-T45) were separated from baseline (FM1-T0), highlighting the rapid response of gut microbial ecosystems to carbohydrate restriction. These changes were not only compositional but also directional: FM1-T90 samples, collected after the LOGI refeeding phase, partially converged towards the LOGI-only group (FM2), suggesting a gradual stabilization of microbial communities following the reinstatement of carbohydrate intake. This convergence may reflect a common trajectory of microbial adaptation under conditions of reduced inflammatory burden and increased metabolic flexibility induced by dietary modulation [26]. At the taxonomic level, FM1 patients showed a progressive enrichment of Firmicutes, a phylum often associated with anti-inflammatory and SCFA-producing taxa such as Faecalibacterium and Roseburia, and a reduction in Proteobacteria, which includes numerous pathobionts linked to intestinal dysbiosis and systemic inflammation (Figure 1 and Figure 7 [27,28]. These findings are consistent with studies demonstrating that ketogenic diets promote eubiosis and mucosal integrity in inflammatory conditions [29,30]. In contrast, FM2 patients, who followed only the LOGI diet, showed greater interindividual variability and a more modest microbial response, strengthening the idea that more significant macronutrient changes are necessary to restore a dysbiotic ecosystem [31]. Fungal dynamics, assessed by 18S rRNA sequencing, revealed complementary profiles. A marked reduction in Ascomycota was observed after the holoprotein phase (T45), followed by a moderate recovery during the subsequent LOGI phase (T90). This partial recovery likely reflects physiological rebalancing rather than pathological fungal overgrowth. At the phylum level, members of the Ascomycota have frequently been implicated in intestinal dysbiosis and chronic inflammatory conditions. Although our analyses did not allow the identification of specific fungal species, previous metabolomic studies in the same FM population have suggested a potential involvement of *Candida albicans* through the modulation of trehalose metabolism, which is linked to cell wall synthesis [32]. Given its known ability to modulate immune responses and alter mucosal homeostasis, the overrepresentation of *Candida albicans* in FM patients may contribute to clinical symptoms [33,34]. In this context, the observed reduction in Ascomycota during the ketogenic phase could reflect a suppression of dysbiosis-associated fungal populations, potentially contributing to the clinical improvements reported in FM1 subjects. The subsequent LOGI diet may act as a stabilizing step, supporting microbial homeostasis without promoting fungal resurgence [35,36]. Overall, these findings suggest that a sequential dietary approach may represent a promising strategy to modulate gut microbiota, potentially influencing opportunistic yeasts, which we hypothesize may include *Candida albicans*, in FM patients. Type 2 FM subjects, in contrast, maintained relatively stable Ascomycota levels across all timepoints, consistent with the moderate glycemic load of the LOGI diet and its role in maintaining fungal balance [37]. These results suggest that fungi, like bacteria, are sensitive to dietary carbohydrate modulation and that transient ketosis may suppress fungal proliferation more effectively than long-term low-glycemic restriction [38,39]. Analysis of the eukaryotic microbiome (Figure 5) also revealed notable fluctuations in minor phyla such as Mucoromycota, Apicomplexa, and Arthropoda, further supporting the idea that diet exerts broad ecological pressure on all microbial kingdoms [40,41]. Although the clinical implications of these changes remain to be fully elucidated, preliminary evidence suggests that these taxes may influence intestinal permeability and immune signaling, particularly in susceptible populations such as FM patients [42,43]. Overall, our findings highlight the diet-responsive nature of the gut microbiome in fibromyalgia at the phylum level. The ketogenic phase was associated with transient changes in microbial composition, including an increase in Verrucomicrobiota and a reduction in Ascomycota, suggesting a potential impact of the dietary intervention on gut microbial communities. The subsequent LOGI diet appears to stabilize these changes, supporting microbial homeostasis over time. These observations indicate that a sequential dietary approach can influence gut microbial composition in fibromyalgia, although the specific functional consequences and effects on individual taxa remain to be elucidated. This study has limitations. The relatively short duration (90 days) and the lack of a healthy control group limit the generalizability of these findings beyond the fibromyalgia population. Moreover, analyses were performed only at the phylum level, and species-level investigations would be required to gain a more detailed understanding of microbial dynamics. Further analyses, including functional profiling (e.g., metagenomics, SCFA quantification), will be necessary to clarify the metabolic and clinical implications of the observed phylum-level changes.

## Data Availability

The original contributions presented in the study are included in the article, further inquiries can be directed to the corresponding authors.
